# Renal Resistive Index in Severe Preeclampsia: Correlation With Glomerular Filtration Rate and Diagnostic Accuracy for Acute Kidney Injury

**DOI:** 10.1155/ijne/9943930

**Published:** 2026-06-03

**Authors:** Jorge Luis Medina-Lopez, Olivia Aleida Cardoso-Navarrete, Job H. Rodriguez-Guillen, Jose J. Zaragoza, Jonathan S. Chavez-Iñiguez

**Affiliations:** ^1^ Critical Care Unit, Women’s Hospital, Morelia, Michoacan, Mexico; ^2^ Mexican Critical Care College, Colegio Mexicano de Medicina Critica, Ciudad de, Mexico; ^3^ Critical Care Department, Hospital H+ Querétaro, Santiago de Querétaro, Mexico; ^4^ Nephrology Service, Hospital Civil de Guadalajara Fray Antonio Alcalde, Guadalajara, Mexico, udg.mx

**Keywords:** acute kidney injury, diagnostic accuracy, glomerular filtration rate, renal resistive index, severe preeclampsia

## Abstract

**Background:**

This study aimed to evaluate the diagnostic performance of renal resistive index (RRI) for predicting acute kidney injury (AKI) and glomerular filtration rate (GFR) in patients with severe preeclampsia.

**Methods:**

This observational study included 22 patients admitted with severe preeclampsia. RRI was measured at admission. AKI was defined and staged according to Kidney Disease: Improving Global Outcomes (KDIGO) criteria. Receiver operating characteristic (ROC) curve analysis was used to assess RRI’s ability to predict AKI and measured creatinine clearance.

**Results:**

Of the 22 patients, 9 (40.9%) developed AKI. Patients who developed AKI had significantly higher admission RRI values compared to those who did not (mean 0.75 ± 0.19 vs. 0.45 ± 0.13, respectively; *p* < 0.001). The area under the ROC curve for RRI predicting AKI was 0.87 (95% CI, 0.65–0.97). In the multivariate linear regression analysis, RRI showed a borderline significant negative association with adjusted GFR (*β* = −112.67; *p* = 0.095), though the overall model was not statistically significant (*p* = 0.23).

**Conclusion:**

The RRI measured at ICU admission is a promising, noninvasive predictor of AKI in patients with severe preeclampsia, demonstrating good diagnostic accuracy with high specificity at an optimal cutoff.

**Summary statement**
 This study demonstrates that the renal resistive index (RRI), obtained by renal Doppler ultrasound, is a useful noninvasive tool for identifying acute kidney injury (AKI) in patients with severe preeclampsia. An RRI threshold of ≥ 0.675 showed high specificity and good overall diagnostic performance. Although the correlation between RRI and glomerular filtration rate did not reach statistical significance, the trend supports the physiological relationship between increased intrarenal vascular resistance and impaired renal function. These findings suggest that bedside assessment of RRI can aid early detection of AKI in preeclamptic women, potentially improving renal monitoring and clinical outcomes.

## 1. Introduction

Preeclampsia is a progressive and unpredictable syndrome complicating a significant percentage of pregnancies globally, posing a serious threat to maternal and fetal health [1]. The impact of this disease is more pronounced in low‐ and middle‐income countries. While many cases are transient, a notable proportion, especially those with early‐onset preeclampsia, can lead to severe or fatal complications for both mother and child. Acute kidney injury (AKI) represents one such critical complication in these patients, and its early detection is paramount for timely intervention and improved outcomes [1].

The renal resistive index (RRI), a noninvasive parameter obtained via Doppler ultrasound, reflects renal hemodynamics and vascular impedance [2, 3]. Its utility as a predictor of AKI has been explored in various general critical care settings; meta‐analyses and prospective studies in critically ill and surgical populations have indicated that an elevated RRI is associated with both the development and persistence of AKI, showing moderate to good predictive performance with reported area under the receiver operating characteristic (ROC) curve (AUC) values around 0.8 and sensitivities and specificities typically ranging from 72% to 79% [4, 5].

Within the context of preeclampsia, the literature suggests that women with this condition often present with higher RRI values compared to those with normotensive pregnancies, reflecting the underlying altered renal hemodynamics and endothelial dysfunction characteristic of the syndrome [6]. While these findings establish RRI as a marker that is altered in preeclampsia and potentially useful for classifying the disease itself, there is limited direct evidence specifically evaluating RRI’s ability to predict the subsequent development of AKI in this unique patient population [7]. Existing meta‐analyses concerning RRI in preeclampsia have predominantly focused on cross‐sectional differences or its role in diagnosing preeclampsia, rather than its capacity for prospective AKI prediction [7].

Therefore, although RRI is a recognized predictor of AKI in general critical care and is known to be elevated in women with preeclampsia [7], its specific utility as an early predictive marker for AKI in patients already diagnosed with preeclampsia remains to be fully elucidated. The present study was designed to investigate the correlation between the RRI and glomerular filtration rate (GFR) in patients with severe preeclampsia, measured upon admission to the intensive care unit (ICU). A further aim was to evaluate the diagnostic performance of RRI as an early marker for AKI in this high‐risk obstetric group and to identify predictors associated with a decline in renal function.

## 2. Methods

### 2.1. Study Design

An observational exploratory (pilot), prospective, correlational, nonexperimental cohort was conducted at the Obstetric ICU of the Women’s Hospital of Morelia, Michoacán, Mexico. This hospital serves as a significant center for obstetric care within the state. The study enrolled women admitted to the obstetric ICU with a diagnosis of severe preeclampsia. Participants were categorized into two groups based on the development of AKI during their ICU stay. Given the absence of prior data specifically evaluating the diagnostic performance of RRI for AKI prediction in severe preeclampsia, no formal a priori sample size calculation was performed. Therefore, the study was designed as hypothesis‐generating.

### 2.2. Inclusion and Exclusion Criteria

Patients were eligible for inclusion if they: (1) were diagnosed with severe preeclampsia upon admission to the obstetric ICU; (2) had a four‐hour timed urine collection initiated on admission for creatinine clearance assessment; and (3) had complete medical records available for review. Patients were excluded if they (1) were admitted to the ICU for reasons other than severe preeclampsia or (2) had incomplete medical records precluding full data extraction.

### 2.3. Data Collection and Definitions

Data were prospectively collected from patient medical records. This included demographic information, obstetric history, baseline clinical measurements, laboratory results, and maternal outcomes.

RRI was determined by a trained operator using Doppler ultrasound, measurements were taken in the renal intralobular arteries, and RRI was calculated using the standard formula: RRI = (peak systolic velocity – end diastolic velocity)/peak systolic velocity [8]. RRI assessment was successfully obtained in all patients included in the study. The operator was not formally blinded to the clinical setting.

Kidney function assessment was made by serum creatinine (sCr) at admission. The primary estimation of the GFR was creatinine clearance adjusted for body surface area (BSA) to 1.73 m^2^ (aGFR). This was calculated from sCr and urinary creatinine (uCr) obtained from a 4 h timed urine collection initiated upon ICU admission. Unadjusted creatinine clearance (CrCl) was also recorded.

AKI diagnosis and staging were based on the Kidney Disease: Improving Global Outcomes (KDIGO) guidelines, considering changes in sCr levels and/or urine output, according to the KDIGO criteria [9].

Preeclampsia was defined as new‐onset hypertension after 20 weeks of gestation [2], characterized by a systolic blood pressure of 140 mm Hg or more, or a diastolic blood pressure of 90 mm Hg or more on two occasions at least 4 h apart, or a systolic blood pressure of 160 mm Hg or more, or a diastolic blood pressure of 110 mm Hg or more, which can be confirmed within minutes. This diagnosis must be accompanied by new‐onset proteinuria, defined as 300 mg or more in a 24 h urine collection, a protein/creatinine ratio of 0.3 or more, or a dipstick reading of 2+. In the absence of proteinuria, new‐onset hypertension with the new onset of any of the following severe features is also diagnostic: thrombocytopenia (platelet count less than $100,000 × 10^9/^L), renal insufficiency (sCr concentration greater than 1.1 mg/dL or a doubling of sCr), impaired liver function (elevated liver transaminases to twice the normal concentration), pulmonary edema, or new‐onset headache unresponsive to medication or visual symptoms [10].

## 3. Primary and Secondary Outcomes

The primary outcome was the diagnostic accuracy of RRI for predicting AKI, assessed using ROC curve analysis. Secondary outcomes included the association between RRI and eGFR and the identification of factors associated with reduced kidney function.

### 3.1. Ethics Approval and Consent to Participate

This study was performed in line with the principles of the Declaration of Helsinki. Approval was granted by the Ethics and Research Committee of the Women’s Hospital of Morelia, reference number HMCEICI/492508. Written informed consent was obtained from all participating patients or their legally authorized representatives prior to their inclusion in the study. Participants received no financial compensation. This study was not funded. Patient confidentiality was maintained by anonymizing all data using numerical codes. All electronic data were stored on a password‐protected computer accessible only to the primary investigator. The study adhered to the Strengthening the Reporting of Observational Studies in Epidemiology (STROBE) guidelines [11].

### 3.2. Statistical Analysis

All analyses were performed in accordance with the exploratory nature of the study. Given the limited sample size, results should be interpreted as hypothesis‐generating. The distribution of continuous variables was assessed for normality using the Shapiro–Wilk test. Normally distributed data were presented as mean ± standard deviation (SD), while non‐normally distributed data were presented as median and interquartile range (IQR). Categorical variables were expressed as frequencies and percentages (*n* (%)).

Comparisons between the AKI and No‐AKI groups were performed using the independent samples *t*‐test for normally distributed continuous variables and the Wilcoxon rank‐sum (Mann–Whitney U) test for non‐normally distributed continuous variables. Pearson’s chi‐squared (*X* ^2^) test or Fisher’s exact test was used for categorical variables, with Fisher’s exact test applied when expected cell counts were small.

ROC curve analysis was conducted to evaluate the ability of RRI to discriminate between patients who developed AKI and those who did not. The area under the ROC curve (AUC) with its 95% confidence interval (CI) was calculated. Sensitivity, specificity, positive predictive value (PPV), negative predictive value (NPV), positive likelihood ratio (LR+), and negative likelihood ratio (LR−) were determined for various RRI cutoff values. The optimal RRI cutoff point for predicting AKI was determined using the Youden index (*J* = sensitivity + specificity−1). Bootstrap resampling (100 replications) was used to estimate the 95% CI of this cutpoint. A Fagan’s nomogram was used to illustrate the clinical utility of the derived likelihood ratios from the optimal RRI cutoff in estimating post‐test probabilities of AKI.

A linear multivariate regression model was performed to identify predictors of aGFR. Variables with a *p*‐value ≤ 0.20 in the bivariate analyses were considered for inclusion in the model, along with RRI. Collinearity was assessed prior to finalizing the model.

To further explore the relationship between renal hemodynamics and renal function, we performed two additional analyses. First, a scatter plot was generated to visualize the association between the RRI and the eGFR. Second, a multiple linear regression model was constructed to determine if RRI was an independent predictor of eGFR as a continuous variable. Based on our bivariate analysis (Table 1), obesity was the only comorbidity with a *p*‐value < 0.20 (*p* = 0.06) and was thus included as a confounder in the final model.

**TABLE 1:** Baseline characteristics of the study population according to AKI status.

Characteristic	No‐AKI (*N* = 13)	AKI (*N* = 9)	Total (*N* = 22)	*p*‐value
*Demographic characteristics*				
Age (years)	28.23 ± 5.40	28.78 ± 7.66	28.45 ± 6.25	0.85
Weight (kg)	80.54 ± 14.89	82.44 ± 20.89	81.32 ± 17.14	0.80
Height (cm)	160.77 ± 5.43	158.89 ± 4.86	160.00 ± 5.17	0.42
BSA (m^2^)	1.89 ± 0.18	1.89 ± 0.23	1.89 ± 0.19	0.97
BMI (kg/m^2^)	31.16 ± 6.66	32.06 ± 7.86	31.53 ± 7.01	0.78

*Hemodynamic and renal parameters*				
Renal resistive index (RRI)	0.45 ± 0.13	0.75 ± 0.19	0.57 ± 0.21	< 0.001^∗^
Mean arterial pressure (MAP, mmHg)	113.62 ± 15.26	108.00 ± 21.78	111.32 ± 17.94	0.48
Creatinine clearance (mL/min)	127.70 ± 59.06	56.01 ± 53.32	98.37 ± 66.17	0.01^∗^
Creatinine clearance/m^2^ (mL/min/1.73 m^2^)	117.54 ± 55.26	49.88 ± 43.47	89.86 ± 60.20	0.01^∗^
Urinary flow (mL)	360 (300–410)	270 (180–550)	355 (232–550)	0.33

*Laboratory parameters*				
Serum creatinine (mg/dL)	0.60 (0.51–0.71)	1.80 (1.49–2.03)	0.72 (0.60–1.50)	< 0.001^∗^
Urinary creatinine (mg/dL)	46 (24–70) (*n* = 13)	42.05 (24.5–84.6) (*n* = 8)	46 (24–70) (*N* = 21)	0.61
Total bilirubin (mg/dL)	0.54 (0.27–0.63)	0.60 (0.20–1.23)	0.57 (0.21–0.86)	0.87
Direct bilirubin (mg/dL)	0.26 (0.12–0.46)	0.25 (0.16–1.11)	0.255 (0.12–0.51)	0.64
Indirect bilirubin (mg/dL)	0.23 (0.16–0.37)	0.12 (0.07–0.35)	0.21 (0.08–0.37)	0.55
AST (U/L)	47 (31–91)	31 (20–265)	44 (23–93)	0.57
ALT (U/L)	48 (27–81)	30 (14–142)	45.5 (16–130)	0.71
Uric acid (mg/dL)	5.93 ± 1.64	6.83 ± 1.13	6.30 ± 1.50	0.17
Cesarean section	12 (92.3%)	7 (77.8%)	19 (86.4%)	0.54

*Comorbidities and pre-existing conditions*				
Chronic arterial hypertension	1 (7.7%)	3 (33.3%)	4 (18.2%)	0.26
Obesity	0 (0.0%)	3 (33.3%)	3 (13.6%)	0.06
Diabetes mellitus	0 (0.0%)	2 (22.2%)	2 (9.1%)	0.16
HELLP syndrome (pre‐existing)	3 (23.1%)	1 (11.1%)	4 (18.2%)	0.62
Fatty liver	0 (0.0%)	1 (11.1%)	1 (4.5%)	0.41
Hyperthyroidism	1 (7.7%)	0 (0.0%)	1 (4.5%)	1.00
Preeclampsia (pre‐existing)	1 (7.7%)	0 (0.0%)	1 (4.5%)	1.00

*Complications during hospitalization*				
Eclampsia	1 (7.7%)	2 (22.2%)	3 (13.6%)	0.54
HELLP syndrome	1 (7.7%)	0 (0.0%)	1 (4.5%)	1.00
Preeclampsia with severe	2 (15.4%)	3 (33.3%)	5 (22.7%)	0.61
Obstetric hemorrhage	0 (0.0%)	1 (11.1%)	1 (4.5%)	0.41

*Note:* Continuous variables were compared using the Wilcoxon rank‐sum (Mann–Whitney) or two‐sample t‐test. Categorical variables were compared using Fisher’s exact test. KDIGO 5 patients (55.6%) were in Stage 1, 2 (22.2%) were in Stage 2, and 2 (22.2%) were in Stage 3. ALT, alanine aminotransferase; AST, aspartate aminotransferase; CrCl, creatinine clearance.

Abbreviations: aGFR = adjusted glomerular filtration rate, AKI = acute kidney injury, BMI = body mass index, BSA = body surface area, and RRI = renal resistive index.

^∗^Statistically significant (*p* < 0.05).

All statistical tests were two‐sided, and a *p*‐value < 0.05 was considered statistically significant. Data were analyzed using Stata Version 16.1 (StataCorp, College Station, TX, USA).

## 4. Results

### 4.1. Patient Characteristics

**FIGURE 1:**
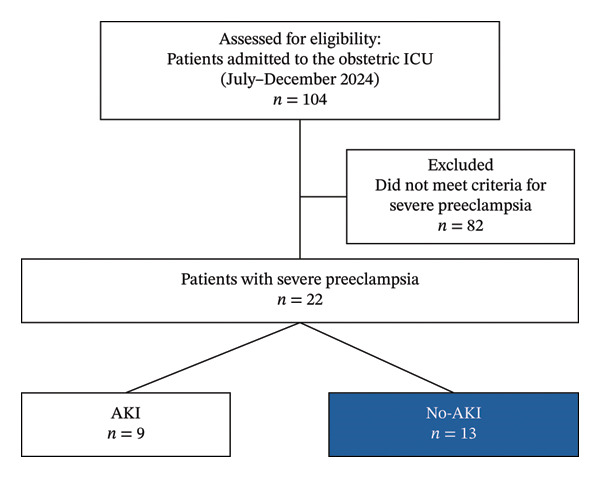
Patient flow diagram.

**FIGURE 2:**
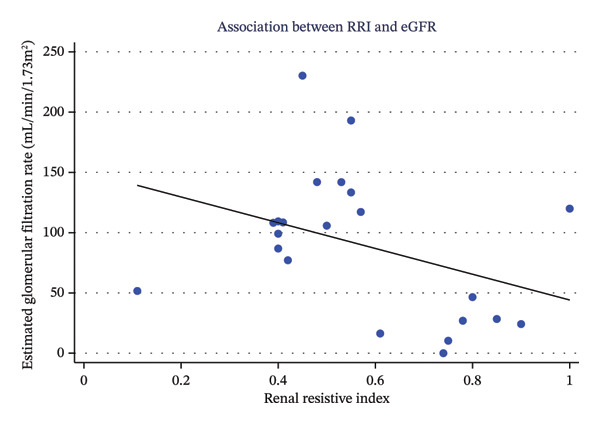
Scatter plot showing the correlation between renal resistive index (RRI) and estimated glomerular filtration rate (eGFR). Higher RRI values tended to associate with lower eGFR, suggesting increased intrarenal vascular resistance in patients with impaired kidney function.

The flow of participants through the study is detailed in Figure 1. Between July 2024 and December 2024, a total of 22 patients were included in the final analysis. Nine patients (40.9%) were classified into the AKI group, and 13 patients (59.1%) were classified into the No‐AKI group. The baseline demographic, hemodynamic, renal, and laboratory characteristics of the study participants, stratified by AKI status, are presented in Table 1. Patients who developed AKI had a significantly higher mean RRI (0.75 ± 0.19 vs. 0.45 ± 0.13; *p* < 0.001), significantly lower mean CrCl (56.01 ± 53.32 mL/min vs. 127.70 ± 59.06 mL/min; *p* = 0.01), and significantly lower mean aGFR (49.88 ± 43.47 mL/min/1.73 m^2^ vs. 117.54 ± 55.26 mL/min/1.73 m^2^; *p* = 0.01) compared to those who did not develop AKI. Furthermore, median sCr was significantly higher in the AKI group (1.80 (1.49–2.03) mg/dL vs. 0.60 (0.51–0.71) mg/dL; *p* < 0.001). Figure 2 shows the correlation between RRI and eGFR. Higher RRI values tended to associate with lower eGFR.

### 4.2. Primary and Secondary Outcomes

**FIGURE 3:**
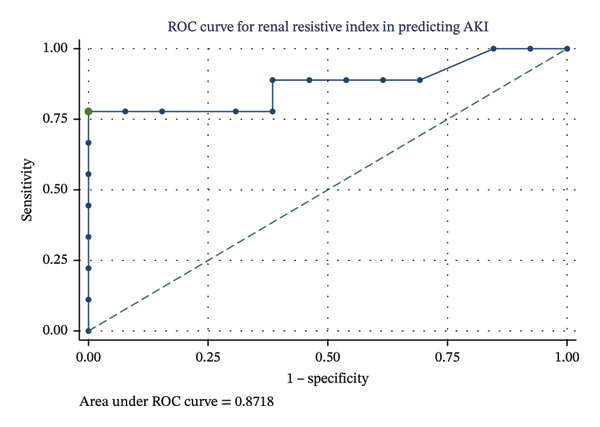
Receiver operating characteristic (ROC) curve for RRI in predicting AKI.

**FIGURE 4:**
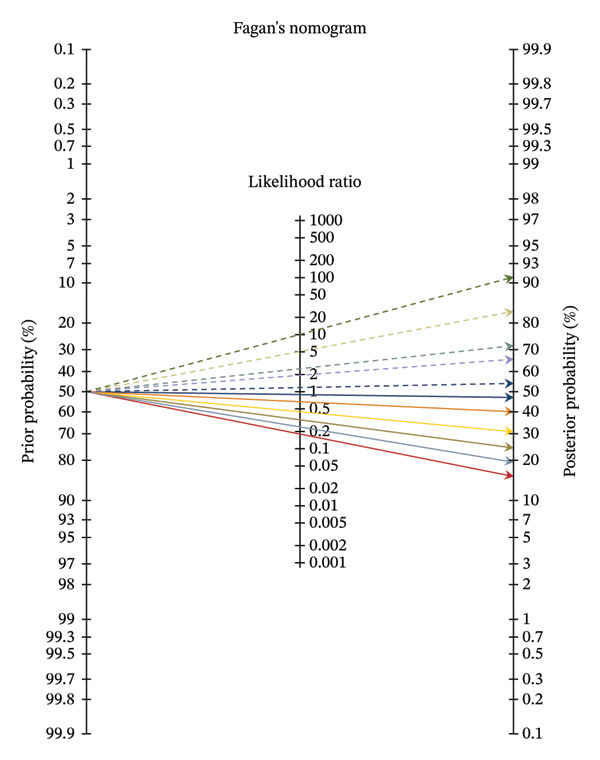
Fagan’s nomogram illustrating the change from pretest to post‐test probability of AKI using the likelihood ratios for the RRI. The Fagan nomogram is presented for illustrative purposes to demonstrate potential clinical application. With a pretest probability of 50%, an RRI ≥ 0.675 (positive likelihood ratio = 3.5) increases the post‐test probability of AKI to approximately 78%, supporting its diagnostic value as a bedside tool.

The ability of RRI to predict AKI was assessed using ROC curve analysis (Figure 3). The area under the ROC curve for RRI in predicting AKI was 0.87 (95% CI, 0.65–0.97), indicating good diagnostic accuracy. Using the Youden index, the optimal cutoff point for RRI was determined to be 0.675, which yielded a sensitivity of 77.8% and a specificity of 100.0%. Bootstrap analysis for this cutoff point yielded a 95% CI of 0.570–0.780. For the optimal RRI cutoff of 0.675, the calculated LR+ is undefined (mathematically infinite) due to the 100% specificity, while the LR− is 0.22. The clinical utility of these likelihood ratios is illustrated using a Fagan’s nomogram (Figure 4). For instance, in a patient with a pretest probability of AKI of 40%, an RRI < 0.675 (negative test, LR− = 0.22) would decrease the post‐test probability of AKI to approximately 13%. Conversely, an RRI ≥ 0.675 (positive test) would substantially increase the post‐test probability, although this should be interpreted cautiously given the sample size. The detailed performance metrics for various RRI cutoff points are presented in Table 2. Our findings suggest that RRI has potential as a rapid, bedside tool to help rule in or rule out AKI in patients with severe preeclampsia, particularly when elevated (≥ 0.675).

**TABLE 2:** Diagnostic performance and optimal cutoff value of the RRI for the prediction of AKI.

RRI cutoff point	Sensitivity (%)	Specificity (%)	LR+	LR−	Youden index
≥ 0.50	88.89	61.54	2.31	0.18	0.50
≥ 0.55	77.78	69.23	2.53	0.32	0.47
≥ 0.57	77.78	84.62	5.06	0.26	0.62
≥ 0.61	77.78	92.31	10.11	0.24	0.70
≥ 0.675	77.78	100.00	∞	0.22	0.78
≥ 0.75	66.67	100.00	∞	0.33	0.67
≥ 0.80	44.44	100.00	∞	0.56	0.44

*Note:* NPV is undefined because specificity is 0, and all nondiseased individuals test positive, so you cannot confidently predict absence of disease. PPV is undefined because sensitivity is 0, and all diseased individuals test negative, so you cannot confidently predict the presence of disease based on a positive test.

Abbreviations: NPV = negative predictive value, PPV = positive predictive value, LR = likelihood ratio.

A multivariate linear regression analysis was performed to identify predictors of eGFR (Table 3). The overall model, which included RRI and obesity, was not statistically significant (*F*(2,19) = 1.60; *p* = 0.23) and explained approximately 14.4% of the variance in eGFR (*R* ^2^ = 0.144). In this model, RRI had a coefficient of −112.67 (95% CI, −247.09 to 21.75), suggesting a negative association with eGFR that was of borderline statistical significance (*p* = 0.095). Obesity was not significantly associated with eGFR (*p* = 0.79). The interpretation of this suggests that renal vascular resistance (as reflected by higher RRI) may contribute to reduced kidney function in severe preeclampsia, although the present sample was underpowered to demonstrate a definitive association.

**TABLE 3:** Multivariable linear regression analysis of factors associated with estimated eGFR.

Variable	Coefficient (*β*)	95% confidence interval	*p*‐value
Renal resistive index (RRI)	−112.67	−247.09 to 21.75	0.095
Obesity (yes vs. no)	10.27	−70.73 to 91.26	0.794
Constant (_cons)	152.94	74.57 to 231.31	0.001

*Model statistics*			
*N*	22		
*F* (2, 19)	1.60		
Prob > *F*	0.2276		
*R*‐squared	0.144		
Adj. *R*‐squared	0.0542		

## 5. Discussion

In this observational study of patients admitted to the ICU with severe preeclampsia, we demonstrated that the RRI may represent a potentially useful tool for renal risk stratification. Our principal findings were threefold: first, RRI measured upon admission was significantly higher in patients who subsequently developed AKI compared to those who did not. Second, RRI showed good diagnostic accuracy for predicting AKI, with an AUC of 0.87. An optimal RRI cutoff of 0.675 was identified, which provided excellent specificity (100.00%) and good sensitivity (77.78%). However, this apparent perfect specificity and the resulting infinite LR+ should be interpreted with caution, as these estimates may be unstable and potentially overestimated due to the small sample size. Third, a higher RRI showed a borderline significant negative association with CrCl adjusted for BSA in a multivariate regression model (*p* = 0.095), directly linking increased intrarenal vascular resistance to diminished GFR.

While our primary analysis robustly demonstrated the utility of RRI as a diagnostic test for predicting AKI using an optimized cutoff, our exploratory analysis using multiple linear regression did not find a statistically significant association between RRI and eGFR as continuous variables. This discrepancy is likely attributable to the limited statistical power of our study due to the small sample size (*n* = 22). A linear regression model with multiple covariates requires a larger cohort to detect significant associations.

Despite the lack of statistical significance in the regression model, the negative trend observed in the scatter plot and the large magnitude of the regression coefficient align with the strong diagnostic performance observed in the ROC analysis. This suggests that a true relationship likely exists, but our study was underpowered to confirm it with this specific statistical approach. Therefore, while the primary strength of RRI in our cohort lies in its ability to classify risk for AKI, future studies with larger sample sizes are warranted to precisely quantify its linear relationship with the decline in GFR.

Our study addresses a critical and previously identified gap in literature. While RRI has been extensively studied in general critical care populations [2–4], there has been a lack of direct evidence evaluating its predictive utility for AKI in pregnancy‐related conditions such as preeclampsia. Previous research on RRI in pregnant women was limited to diagnosing acute ureteral obstruction, a distinct pathophysiology from the intrinsic AKI of preeclampsia [12]. To our knowledge, this is the first study to specifically assess the diagnostic accuracy of RRI for AKI in a cohort of women with severe preeclampsia, providing novel and clinically relevant data.

The diagnostic performance of RRI in our cohort (AUC = 0.87) was robust, exceeding the pooled AUC of ∼0.83 reported in meta‐analyses of heterogeneous critically ill populations [2, 3]. However, these findings should be interpreted with caution given the small sample size, which may lead to overestimation of accuracy, potential overfitting, and instability of the identified cutoff value, standing in contrast to the more modest performance (AUC < 0.6) found in some large, multicenter studies of unselected ICU patients [13, 14]. This suggests that RRI may be a particularly powerful biomarker in specific, pathologically homogeneous subgroups. In severe preeclampsia, where systemic endothelial dysfunction and renal vasoconstriction are central pathophysiological mechanisms [6], RRI appears to be an exceptionally well‐suited tool to capture the severity of renal hemodynamic compromise.

A key finding is our identified optimal RRI cutoff of 0.675, which yielded a specificity of 100%. This cutoff is remarkably similar to the ∼0.685 threshold used to distinguish persistent from transient AKI in other ICU settings [5] and the ≥ 0.70 threshold associated with increased all‐cause mortality in patients with CKD [15]. The convergence of these values is provocative; it suggests that the level of intrarenal vascular resistance that signals impending AKI in severe preeclampsia is in the same range as that which indicates severe, chronic renovascular disease and long‐term adverse outcomes in other vasculopathic conditions [15–17]. This reinforces the concept that RRI in our patients is likely a marker of the severity of their systemic vascular disease, a notion supported by kidney transplant literature where RRI often reflects the recipient’s systemic vascular health rather than isolated graft pathology [18, 19].

The clinical implications of our findings are significant, especially for clinical practice in low‐ and middle‐income countries, where the burden of preeclampsia is highest. RRI is a noninvasive, rapid, relatively low‐cost, and readily available bedside tool. It offers a practical alternative to more expensive, less accessible, or invasive diagnostics, such as novel urinary biomarkers (e.g., NGAL and NephroChek) or kidney biopsy [2, 5, 8, 20].

Clinically, RRI could be integrated into a risk stratification protocol for women admitted with severe preeclampsia. An RRI ≥ 0.675, with its 100% specificity in our cohort, acts as a strong “rule‐in” test for high AKI risk, justifying heightened surveillance and proactive management. As illustrated with Fagan’s nomogram (Figure 4), this result can dramatically increase the post‐test probability of AKI. Conversely, an RRI < 0.675 (LR− = 0.22) substantially lowers the post‐test probability, providing valuable reassurance and potentially preventing unnecessary interventions. This approach could guide decisions on fluid management, avoidance of nephrotoxic agents, and timing of delivery.

This study has several strengths. It addresses a specific and clinically relevant question regarding AKI prediction in a high‐risk obstetric population (severe preeclampsia), a gap identified in the current literature. The use of RRI offers a noninvasive approach to assess renal risk. Furthermore, we determined an optimal RRI cutoff with high specificity for AKI in this cohort and reported detailed diagnostic performance metrics.

However, certain limitations must be acknowledged. The primary limitation is the small sample size (*N* = 22), which restricts the statistical power of our analyses, particularly for the multivariate regression model, and may lead to wider confidence intervals for our estimates. Consequently, the findings, especially the specific RRI cutoff value and regression coefficients, should be considered preliminary and require validation in larger cohorts.

Second, this was a single‐center study, and thus, the findings may not be generalizable to other populations or settings with different patient characteristics or management protocols. Third, the observational nature of the study means we can identify associations but cannot establish causality between RRI and AKI development; selection bias cannot be excluded, which may limit the representativeness of the study population. Fourth, while RRI measurement was performed by trained personnel, inter‐observer variability, a general concern with ultrasound‐based measurements, cannot be entirely excluded, although this was not specifically assessed in our study. Finally, GFR was estimated using CrCl rather than measured by a gold‐standard technique, though CrCl is a commonly accepted clinical method. Despite these limitations, our study provides a strong foundation for future, larger multicenter studies to validate these findings.

These promising results should serve as a foundation for future research. There is a clear need for larger, multicenter prospective studies to validate the RRI cutoff of 0.675 for AKI prediction in women with severe preeclampsia. Future studies should also assess the value of serial RRI measurements for monitoring disease progression. Finally, investigating the link between admission RRI during a preeclamptic pregnancy and the long‐term risk of cardiovascular and CKD would be a valuable avenue, given the established role of RRI as a prognostic marker in other chronic vascular diseases [15–17, 21].

## 6. Conclusion

This study provides novel hypothesis‐generating evidence that the RRI may represent a promising, noninvasive tool for early risk stratification for the early prediction of AKI in patients with severe preeclampsia, although these findings require validation in larger studies. An RRI value ≥ 0.675 demonstrated excellent specificity and good overall diagnostic accuracy. Given its accessibility and low cost, RRI holds significant potential to enhance risk stratification and guide clinical management for this vulnerable patient population, particularly in resource‐limited settings. Further validation in larger studies is essential to confirm these findings and facilitate the integration of RRI into standard clinical practice.

**Nomenclature**
AKI: Acute kidney injury
AUC: Area under the curve
BMI: Body mass index
CI: Confidence interval
eGFR: Estimated glomerular filtration rate
HELLP: Hemolysis, elevated liver enzymes, and low platelet count
ICU: Intensive care unit
IQR: Interquartile range
KDIGO: Kidney Disease: Improving Global Outcomes
LR+: Positive likelihood ratio
LR−: Negative likelihood ratio
MAP: Mean arterial pressure
NPV: Negative predictive value
PPV: Positive predictive value
ROC: Receiver operating characteristic
RRI: Renal resistive index
SD: Standard deviation

## Author Contributions

The contributions of the authors were as follows: The study was conceptualized by Jorge Luis Medina‐Lopez (JLML), Olivia Aleida Cardoso‐Navarrete (OACN), and Jose J. Zaragoza (JJZ). The methodology was developed by Jorge Luis Medina‐Lopez, Jonathan S. Chavez‐Iñiguez, and Jose J. Zaragoza. The formal analysis and data curation were performed by Jose J. Zaragoza and Job H. Rodriguez‐Guillen (JHRG). The investigation and provision of resources were carried out by Jorge Luis Medina‐Lopez, Olivia Aleida Cardoso‐Navarrete, and Job H. Rodriguez‐Guillen. The original draft was written by Jorge Luis Medina‐Lopez and Jose J. Zaragoza. All authors (Jorge Luis Medina‐Lopez, Olivia Aleida Cardoso‐Navarrete, Job H. Rodriguez‐Guillen, Jonathan S. Chavez‐Iñiguez, and Jose J. Zaragoza) participated in the critical review and editing of the manuscript. Project administration was handled by Jorge Luis Medina‐Lopez, Olivia Aleida Cardoso‐Navarrete, and Jose J. Zaragoza. All authors have read and approved the final version of the manuscript.

## Funding

No funding was received for this manuscript.

## Ethics Statement

This study was performed in line with the principles of the Declaration of Helsinki. Approval was granted by the Ethics and Research Committee of the Women’s Hospital of Morelia, reference number HMCEICI/492508. Written informed consent was obtained from all participating patients or their legally authorized representatives prior to their inclusion in the study. Participants received no financial compensation. This study was not funded. Patient confidentiality was maintained by anonymizing all data using numerical codes. All electronic data were stored on a password‐protected computer accessible only to the primary investigator.

## Consent

The authors have nothing to report.

## Conflicts of Interest

The authors declare no conflicts of interest.

## Data Availability

The data that support the findings of this study are not openly available due to patient confidentiality and privacy regulations. Data may be available from the corresponding author upon reasonable request and subject to approval from the institutional ethics committee.
